# Integrating single-cell and bulk RNA sequencing data unveils antigen presentation and process-related CAFS and establishes a predictive signature in prostate cancer

**DOI:** 10.1186/s12967-023-04807-y

**Published:** 2024-01-14

**Authors:** Wenhao Wang, Tiewen Li, Zhiwen Xie, Jing Zhao, Yu Zhang, Yuan Ruan, Bangmin Han

**Affiliations:** Department of Urology, School of Medicine, Shanghai General Hospital, Shanghai Jiao Tong University, Shanghai, 200080 China

## Abstract

**Background:**

Cancer-associated fibroblasts (CAFs) are heterogeneous and can influence the progression of prostate cancer in multiple ways; however, their capacity to present and process antigens in PRAD has not been investigated. In this study, antigen presentation and process-related CAFs (APPCAFs) were identified using bioinformatics, and the clinical implications of APPCAF-related signatures in PRAD were investigated.

**Methods:**

SMART technology was used to sequence the transcriptome of primary CAFs isolated from patients undergoing different treatments. Differential expression gene (DEG) screening was conducted. A CD4 + T-cell early activation assay was used to assess the activation degree of CD4 + T cells. The datasets of PRAD were obtained from The Cancer Genome Atlas (TCGA) database and NCBI Gene Expression Omnibus (GEO), and the list of 431 antigen presentation and process-related genes was obtained from the InnateDB database. Subsequently, APP-related CAFs were identified by nonnegative matrix factorization (NMF) based on a single-cell seq (scRNA) matrix. GSVA functional enrichment analyses were performed to depict the biological functions. A risk signature based on APPCAF-related genes (APPCAFRS) was developed by least absolute shrinkage and selection operator (LASSO) regression analysis, and the independence of the risk score as a prognostic factor was evaluated by univariate and multivariate Cox regression analyses. Furthermore, a biochemical recurrence-free survival (BCRFS)-related nomogram was established, and immune-related characteristics were assessed using the ssGSEA function. The immune treatment response in PRAD was further analyzed by the Tumor Immune Dysfunction and Exclusion (TIDE) tool. The expression levels of hub genes in APPCAFRS were verified in cell models.

**Results:**

There were 134 upregulated and 147 downregulated genes, totaling 281 differentially expressed genes among the primary CAFs. The functions and pathways of 147 downregulated DEGs were significantly enriched in antigen processing and presentation processes, MHC class II protein complex and transport vesicle, MHC class II protein complex binding, and intestinal immune network for IgA production. Androgen withdrawal diminished the activation effect of CAFs on T cells. NMF clustering of CAFs was performed by APPRGs, and pseudotime analysis yielded the antigen presentation and process-related CAF subtype CTSK + MRC2 + CAF-C1. CTSK + MRC2 + CAF-C1 cells exhibited ligand‒receptor connections with epithelial cells and T cells. Additionally, we found a strong association between CTSK + MRC2 + CAF-C1 cells and inflammatory CAFs. Through differential gene expression analysis of the CTSK + MRC2 + CAF-C1 and NoneAPP-CAF-C2 subgroups, 55 significant DEGs were identified, namely, APPCAFRGs. Based on the expression profiles of APPCAFRGs, we divided the TCGA-PRAD cohort into two clusters using NMF consistent cluster analysis, with the genetic coefficient serving as the evaluation index. Four APPCAFRGs, THBS2, DPT, COL5A1, and MARCKS, were used to develop a prognostic signature capable of predicting BCR occurrence in PRAD patients. Subsequently, a nomogram with stability and accuracy in predicting BCR was constructed based on Gleason grade (p = n.s.), PSA (p < 0.001), T stage (p < 0.05), and risk score (p < 0.01). The analysis of immune infiltration showed a positive correlation between the abundance of resting memory CD4 + T cells, M1 macrophages, resting dendritic cells, and the risk score. In addition, the mRNA expression levels of THBS2, DPT, COL5A1, and MARCKS in the cell models were consistent with the results of the bioinformatics analysis.

**Conclusions:**

APPCAFRS based on four potential APPCAFRGs was developed, and their interaction with the immune microenvironment may play a crucial role in the progression to castration resistance of PRAD. This novel approach provides valuable insights into the pathogenesis of PRAD and offers unexplored targets for future research.

**Supplementary Information:**

The online version contains supplementary material available at 10.1186/s12967-023-04807-y.

## Introduction

Prostate cancer is the most prevalent cancer in males worldwide and the second leading cause of cancer-related death in men [1]. Due to the paramount role of androgen receptor (AR) signaling in prostate cancer cell growth and survival in the regulation of cell growth and survival [2–4], androgen deprivation therapy (ADT), which inhibits AR signaling, has been the standard treatment for early-stage and metastatic prostate cancer until recently [5, 6]. Unfortunately, after 18–24 months of ADT, including the recently developed potent antiandrogen enzalutamide (Enz), most patients eventually relapse and develop castration-resistant prostate cancer (CRPC) [7–9]. In addition to endocrine therapy, new drugs [11, 12] have been investigated and developed in recent years, and chemotherapy can be used as an alternative treatment for patients who have failed endocrine therapy [10], although its efficacy for CRPC patients is still poor. Moreover, there are currently no curative treatment options for metastatic castration-resistant prostate cancer (mCRPC), and its prognosis is dismal [13]. Therefore, it is imperative to elucidate the underlying mechanisms of castration resistance after ADT in prostate cancer.

The tumor microenvironment (TME) is a highly complex system composed of tumor cells and stromal cells, resulting in the multifaceted nature of malignant tumors [14]. Traditionally, the primary focus in comprehending carcinogenesis has been the tumor cell and its underlying mechanisms [15]. However, dynamic cross-talk between cancer cells and stromal cells is also crucial for cancer progression [16–18]. CAFs, which are activated fibroblasts, are components of the tissue microenvironment [19]. The stromal-to-tumor interaction is largely influenced by CAFs, which stand as the most prominent stromal component within the TME [20]; they can secrete cytokines, chemokines, and growth factors that exert direct and indirect effects on tumorigenesis, proliferation, progression, and invasion of cancer cells [21–23]. At the same time, novel therapies targeting cancer, with mechanisms such as interfering with tumor metabolism to inhibit its malignant progression [24], treat tumors through targeted nanomedicine delivery systems [25, 26]. Moreover, progress in drug delivery using nanoparticles has led to substantial improvements in the efficiency of delivering drugs to disease sites, consequently greatly enhancing therapeutic effectiveness [27–30]. In contrast to traditional endocrine therapy drugs, nanoparticles efficiently direct anti-cancer medications towards metastatic sites of prostate cancer while minimizing adverse effects on the host [31, 32]. In the development and application of these therapies, CAFs exhibit important activities. As with the diversity in CAF origins, the heterogeneity in CAF fate and function has received great attention and has led to the possibility of targeting a subpopulation of CAFs to combat cancer. The common classification of CAFs is myofibroblastic CAFs (myCAFs) and inflammatory CAFs (iCAFs) [33, 34]. Antigen-presenting CAFs (apCAFs), which present MHC class-II-restricted antigens and activate CD4 T cells to demonstrate antigen-presenting capacities, were identified and reported for the first time in pancreatic cancer with the efficacy of single-cell transcriptomics [35]. Nevertheless, the comprehensive characteristics of apCAFs and their associations with prognosis and immunotherapy response in prostate cancer remain inadequately understood.

In this study, we utilized single-cell RNA-sequencing (scRNA-seq) data and transcriptome data to identify subclusters of APPCAFs and develop an APPCAF-associated risk signature for PRAD. In addition, we analyzed the clinical characteristics associated with the APPCAF signature and investigated the immune landscape and immunotherapy responsiveness. Furthermore, using the risk score and clinicopathological characteristics, we developed a prognostic nomogram to analyze the correlation between APPCAF characteristics and PRAD prognosis. Our findings provide novel microenvironment insight into the pathophysiology of PRAD and may provide ideas and approaches for treating prostate cancer.

## Results

### Isolation and transcriptomic profiling of CAFs from patients with PCa

**Fig. 1:**
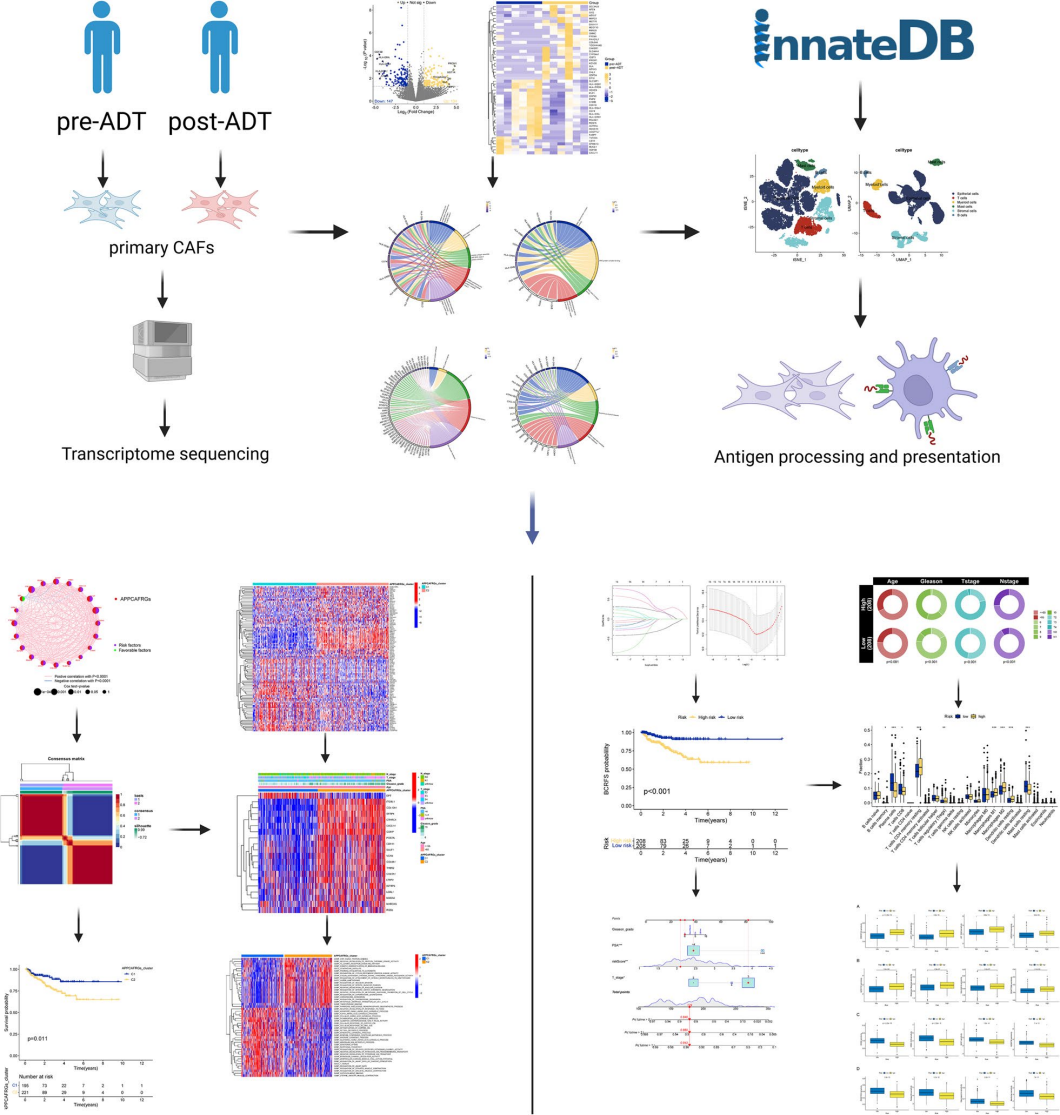
Study flow diagram

**Fig. 2:**
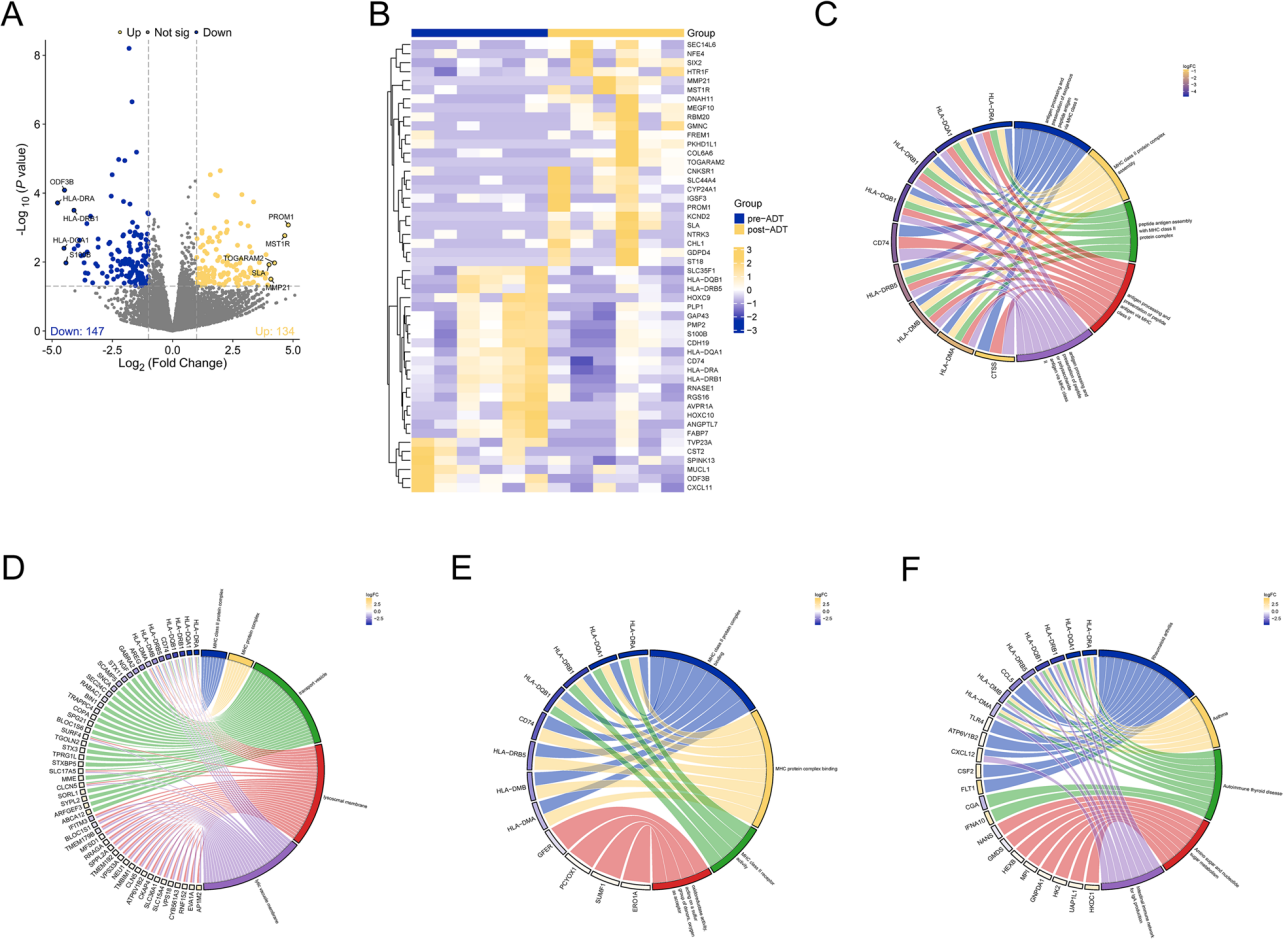
Identification of DEGs between pre-ADT and post-ADT treatments through transcriptomic sequencing data analysis and functional enrichment analysis. **A** Volcano diagram of DEGs. **B** Heatmap of transcriptomic data. Enrichment analysis of DEGs in GO-BP terms (**C**), GO-CC terms (**D**), GO-MF terms (**E**), and KEGG pathways (**F**)

Figure 1 depicts the framework and experimental procedures of the entire study. To elucidate the differential gene expression profiles between the pre-ADT and post-ADT groups, transcriptome sequencing was performed. Based on our transcriptome sequencing data, a total of 281 differentially expressed genes (DEGs) were identified between the pre-ADT and post-ADT groups, including 134 upregulated genes and 147 downregulated genes (Fig. 2A). The heatmap of DEGs revealed distinct clustering of samples from the pre-ADT and post-ADT groups (Fig. 2B). Subsequently, enrichment analysis was conducted on the identified DEGs. The DEGs were significantly enriched in GO-BP terms, such as antigen processing and presentation processes (Fig. 2C); GO-CC terms, such as MHC class II protein complex and transport vesicle (Fig. 2D); GO-MF terms, such as MHC class II protein complex binding (Fig. 2E); and KEGG signaling pathways, such as intestinal immune network for IgA production (Fig. 2F). These results indicated a significant decrease in CAFs implicated in APP following ADT treatment.

### Identification of APP-related CAFs contributing to the TME in PCa

**Fig. 3:**
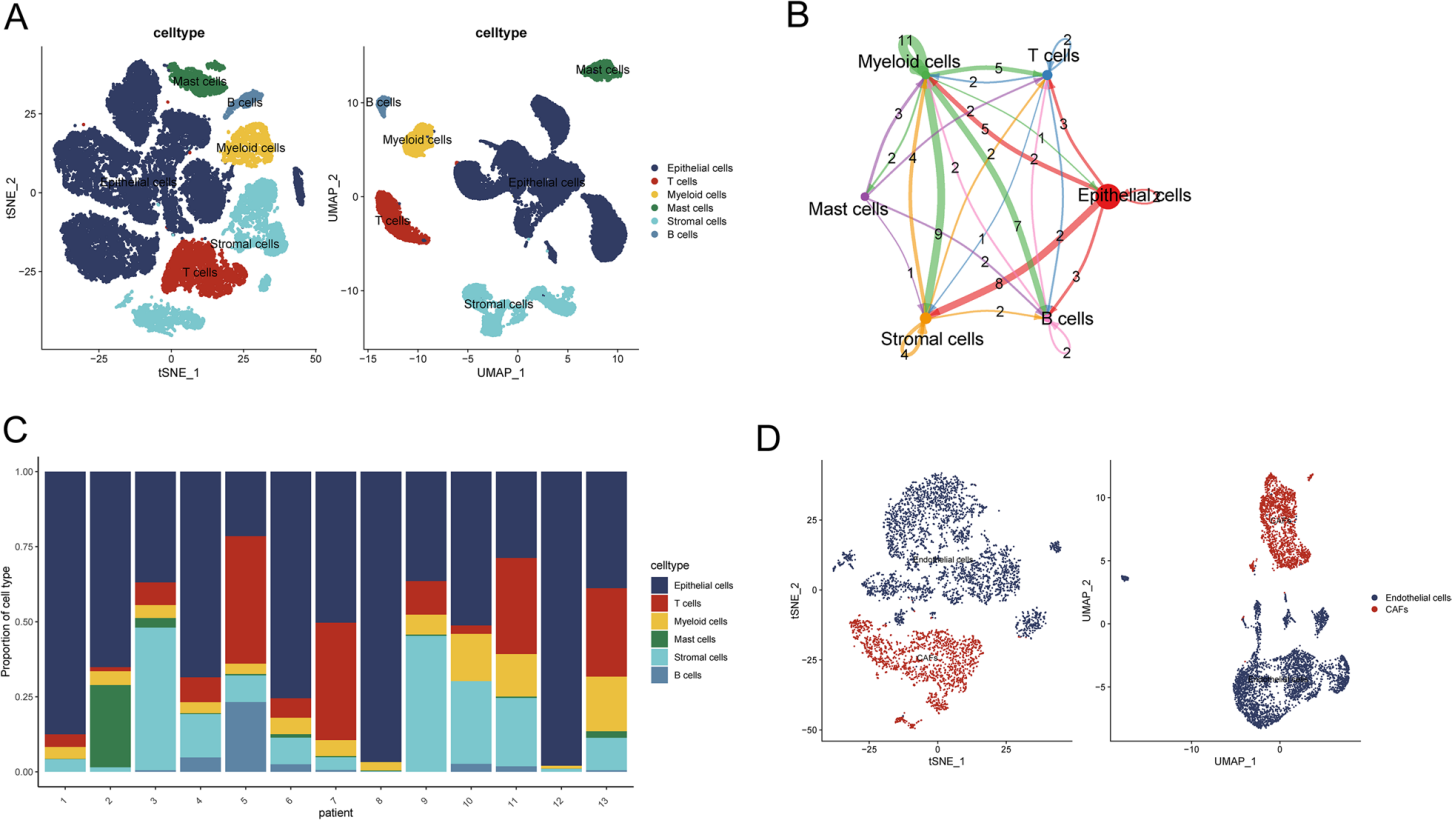
Overview of prostate cancer scRNA-seq from GSE141445. **A** The cell type annotation of 32,602 cells using t-distributed stochastic neighbor embedding (t-SNE) and uniform manifold approximation and projection (UMAP) plots. **B** Analysis of cell‒cell communication between six major cell types using CellChat. **C** Cellular composition across patients, showing the distribution of cell types. **D** Identification of CAFs from the stromal cells

**Fig. 4:**
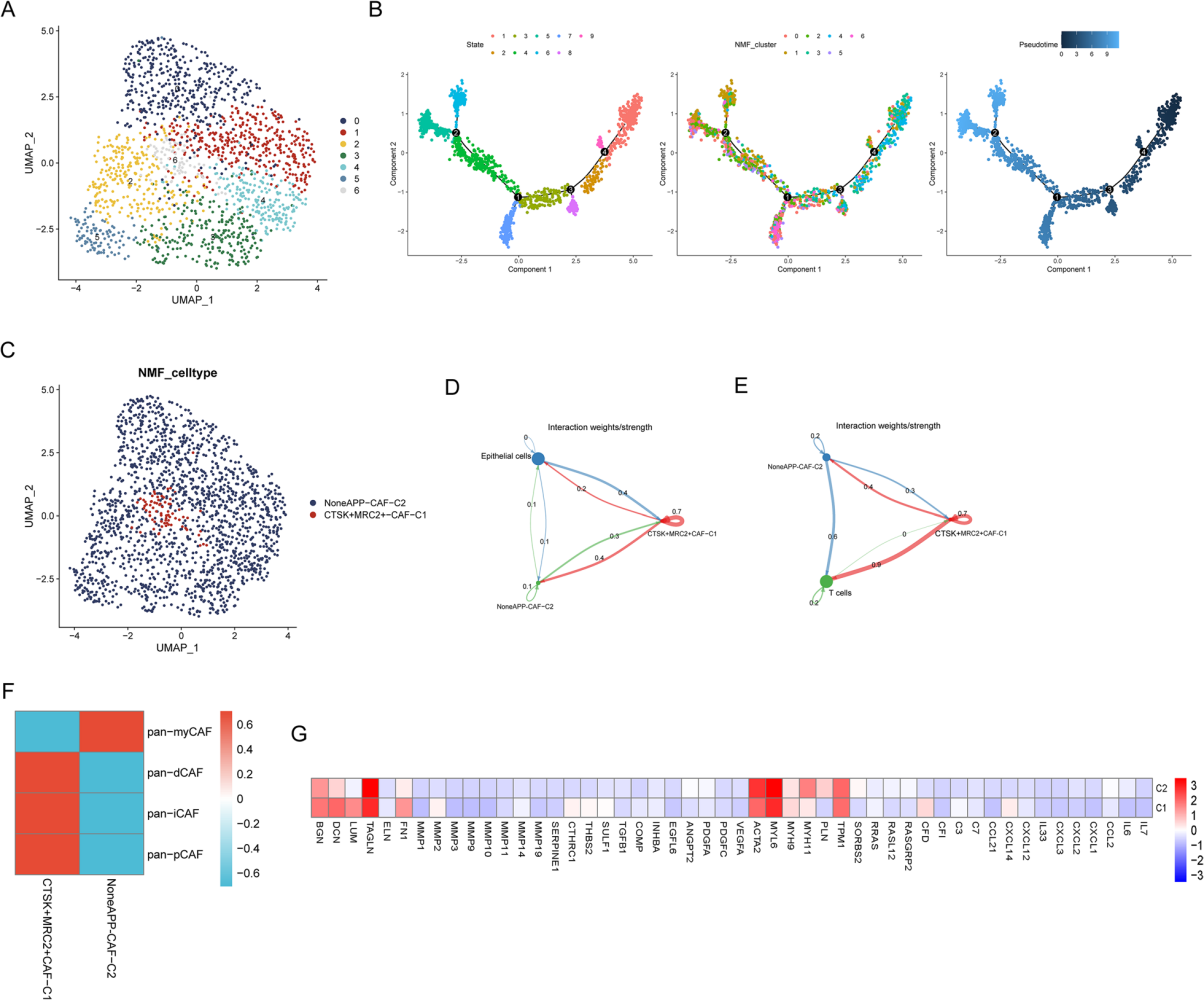
Identification of antigen processing and presentation-related subtypes in CAFs. **A** UMAP visualization of NMF clustering subtypes based on APP-related genes in CAFs. **B** Pseudotime trajectory analysis of NMF clusters. **C** Definition of APP-related subtypes in CAFs. Cell‒cell communication from APP-related CAFs to epithelial cells (**D**) and T cells (**E**). **F** Associations between APP-related CAF subtypes and previous signatures. **G** Heatmap demonstrating differential average expression levels of common signaling pathway genes between the two APP-related subtypes

The PCa scRNA-seq used in our study consisted of 36,424 cells from 13 samples of prostate cancer patients, with major cell types such as epithelial cells, T cells, myeloid cells, stromal cells, and B cells annotated (Fig. 3A). The analysis of cellChat unveiled a myriad of interactions among these cellular types (Fig. 3B). The proportions of the six cell types across the 13 prostate cancer samples are shown in Fig. 3C. Within the PCa dataset, stromal cells were classified into CAFs and endothelial cells (Fig. 3D). Subsequently, NMF clustering of CAFs was performed using APPRGs, resulting in the identification of seven subtypes (Fig. 4A). Next, pseudotime analysis displayed the trajectories of NMF-clustered CAF subtypes (Fig. 4B). Further analysis of the feature genes for the seven NMF subtypes revealed APP-related CAFs, namely, CTSK + MRC2 + CAF-C1. CAFs that did not exhibit APP-related effects were designated as NoneAPP-CAF-C2 (Fig. 4C). In addition, cellChat analysis demonstrated that compared to Non-APP-CAF-C2 cells, CTSK + MRC2 + CAF-C1 cells exhibited more ligand‒receptor connections with epithelial cells and T cells (Fig. 4D, E). Additionally, by calculating the Pan-CAF score based on previously reported signatures [36], we found a strong association between CTSK + MRC2 + CAF-C1 cells and inflammatory CAFs (Fig. 4F). As shown in Fig. 4G, various genes related to the ECM, MMPs, and proinflammatory processes were significantly upregulated in CTSK + MRC2 + CAF-C1 cells.

### Identification of APPCAF-related genes using single-cell RNA-sequencing data

**Fig. 5:**
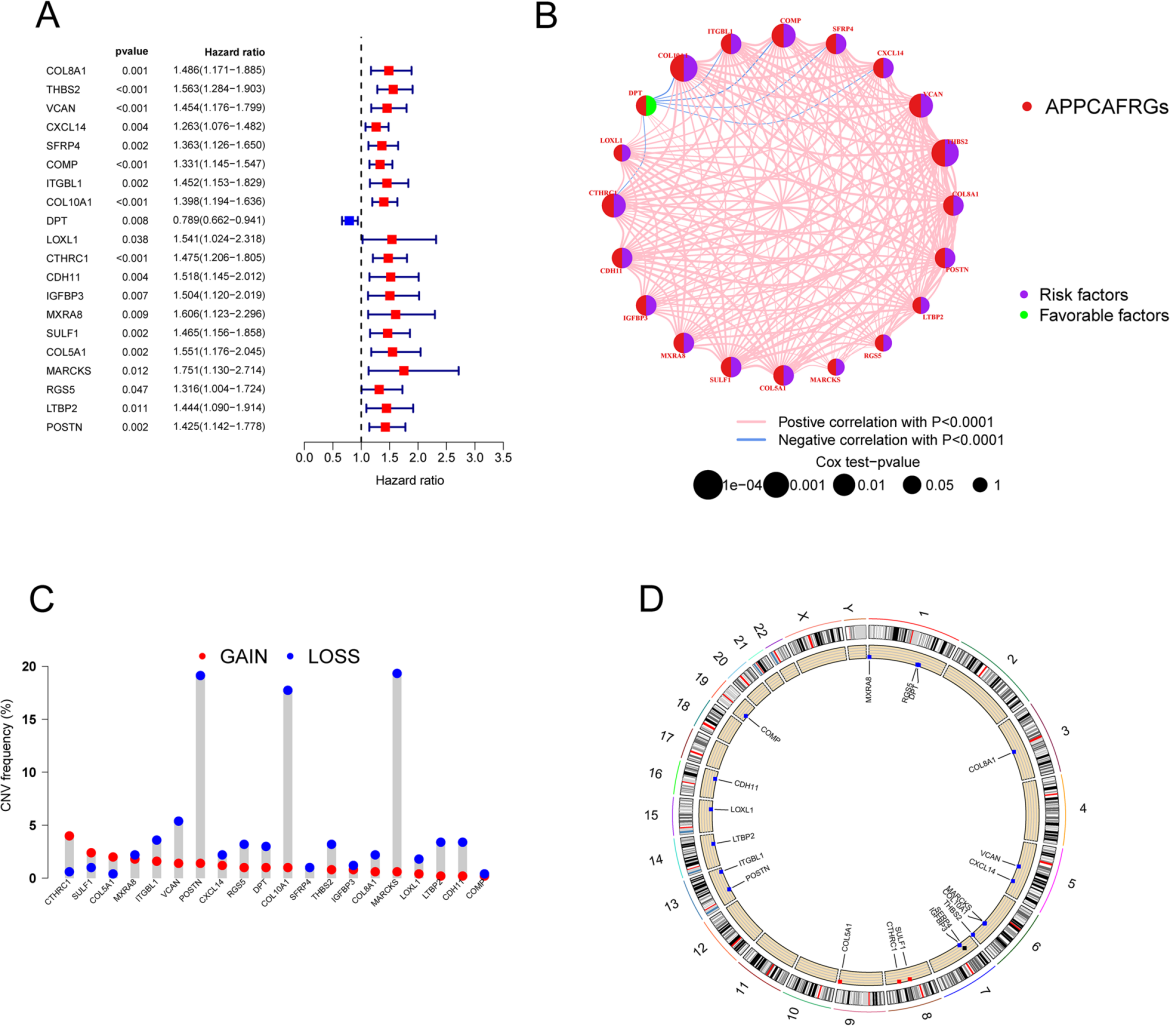
Analysis of characteristic APPCAF-related genes from scRNA-seq. **A** Univariate Cox regression analysis conducted on 55 APPCAF-related genes in the TCGA-PRAD cohort. **B** Circos plot illustrating the correlations of 20 prognosis-associated APPCAF-related genes. **C** Copy number variation (CNV) frequency analysis of 20 APPCAF-related genes. **D** Chromosomal localization of the 20 APPCAF-related genes

Differential gene expression analysis of the CTSK + MRC2 + CAF-C1 and NoneAPP-CAF-C2 subgroups revealed 55 significantly differentially expressed genes, which were designated APPCAF-related genes (APPCAFRGs) (Additional file 3: Table S3). Subsequently, through univariate Cox regression analysis, we identified 20 genes with prognostic value for BCR (Fig. 5A). The correlation circus plot in Fig. 5B depicts the relationships among these genes. Furthermore, analysis of copy number variation (CNV) rates revealed a high frequency of deletions in POSTN, COL10A1, and MARCKS in TCGA (Fig. 5C). The genomic loci of these genes on human chromosomes are illustrated in Fig. 5D.

### NMF Clustering Analysis Based on TCGA-PRAD Patients

**Fig. 6:**
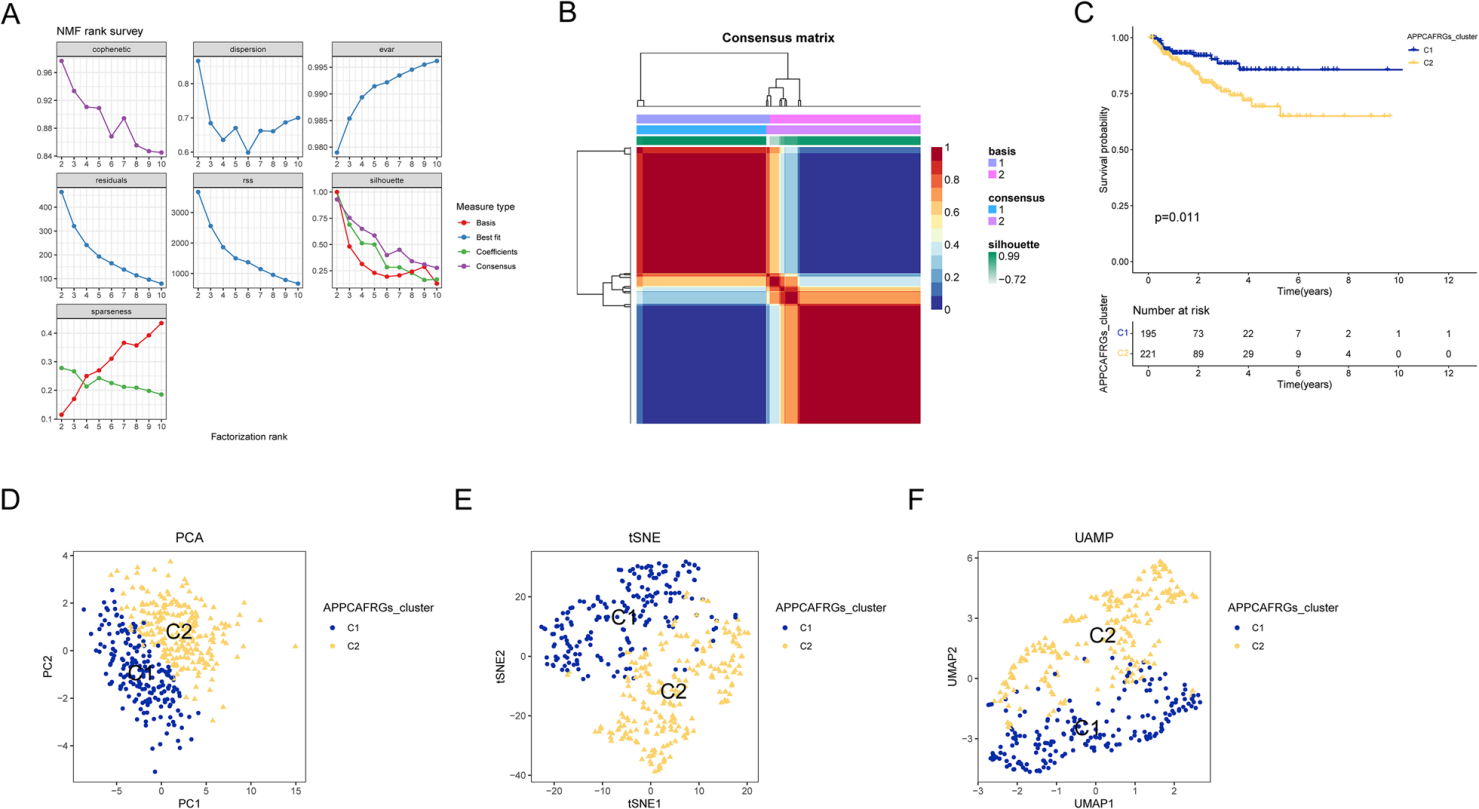
NMF clustering divided the samples in the TCGA-PRAD cohort into two subgroups. **A** Rank and cophenetic correlation coefficients after NMF rank survey. **B** A consensus map of NMF clusters. **C** KM curve for the clusters based on APPCAF-related genes. **D** Scatter plot of PCA scores of two APPCAFRG clusters. **E** tSNE plot of the two clusters. **F** UMAP plot of the two clusters

Next, we performed NMF consensus clustering analysis using the expression profiles of the 20 APPCAFRGs. As depicted in Fig. 6A and B, we successfully partitioned the TCGA-PRAD cohort into two clusters and optimized the grouping using the cophenetic coefficient as an evaluation metric. Subsequently, we assessed the prognostic disparities between the clusters using KM curve analysis and found a significant distinction in patient outcomes between the C1 and C2 subtypes (p = 0.011). Additionally, patients within the C2 cluster exhibited a markedly shorter median time to BCR (Fig. 6C). Moreover, to further investigate the distinctive characteristics, we applied PCA, tSNE, and UMAP dimensionality reduction techniques, which unequivocally demonstrated significant discrepancies between the C1 and C2 subtypes (Fig. 6D).

### Characteristics of APPCAF-related gene subtypes in PCa patients

**Fig. 7:**
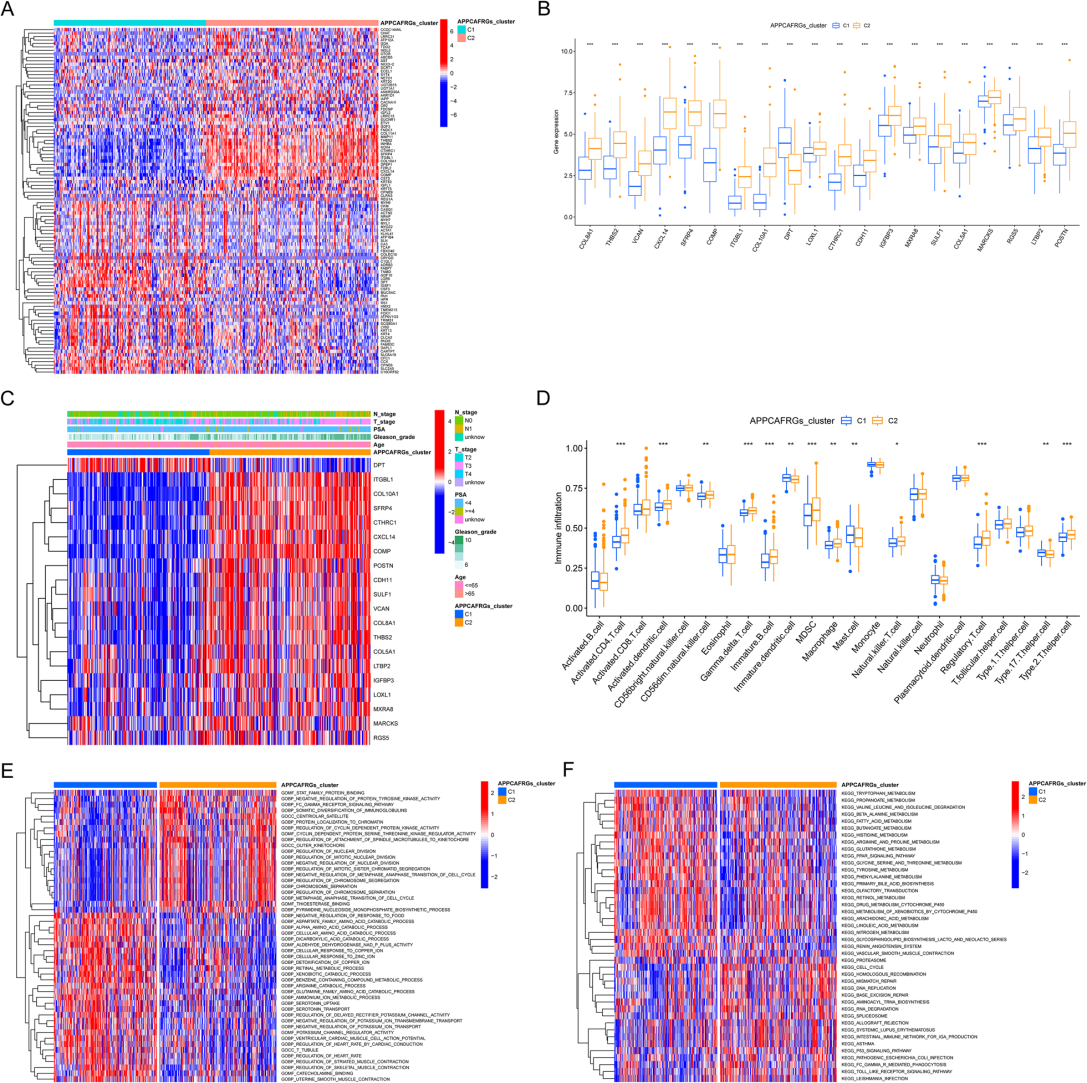
Comparative analysis of clinical characteristics, tumor immune microenvironment, and gene enrichment among distinct subtypes derived from NMF clustering. **A** Heatmap depicting the DEGs among the subtypes identified through NMF clustering. **B** Expression patterns of 20 prognosis-associated APPCAF-related genes across the subtypes. **C** Comparison of clinical and pathological factors between the two subtypes. **D** Differential analysis of 23 immune cell types between the two clusters. Enrichment analysis of DEGs between the two subtypes in GO (**E**) and KEGG pathways (**F**)

We performed gene differential expression analysis between the C1 and C2 subtypes, and the top 50 significant DEGs are presented in Fig. 7A. Subsequently, we investigated the expression patterns of the 20 APPCAF-related genes in the C1 and C2 subtypes. Consistent with the findings from the univariate Cox analysis, genes with HR > 1 exhibited higher expression levels in the C2 subtype, while genes with HR < 1 showed higher expression levels in the C1 subtype (Fig. 7B). The distribution of various clinical features in the C1 and C2 subtypes within the TCGA-PRAD samples is depicted in Fig. 7C. Furthermore, we assessed the proportions of 23 tumor immune cell infiltrations in the C1 and C2 subtypes utilizing the ssGSEA method (Fig. 7D). In addition, we performed GO and KEGG enrichment analyses to identify significant pathways and functions linked to the DEGs between the C1 and C2 subtypes. The KEGG enrichment analysis revealed significant enrichment of pathways such as tryptophan metabolism, propanoate metabolism, and alanine, leucine, and isoleucine degradation in the C1 subtype. In contrast, the C2 subtype exhibited significant enrichment in pathways related to the cell cycle, homologous recombination, and DNA mismatch repair (Fig. 7E). The GO enrichment analysis showed significant enrichment of the C1 subtype in various amino acid catabolic processes, while the C2 subtype exhibited significant enrichment in pathways related to STAT protein family binding, negative regulation of tyrosine kinase activity, and Fcγ receptor signaling (Fig. 7F).

### Establishment and validation of the prognostic signatures of APPCAF-related genes

**Fig. 8:**
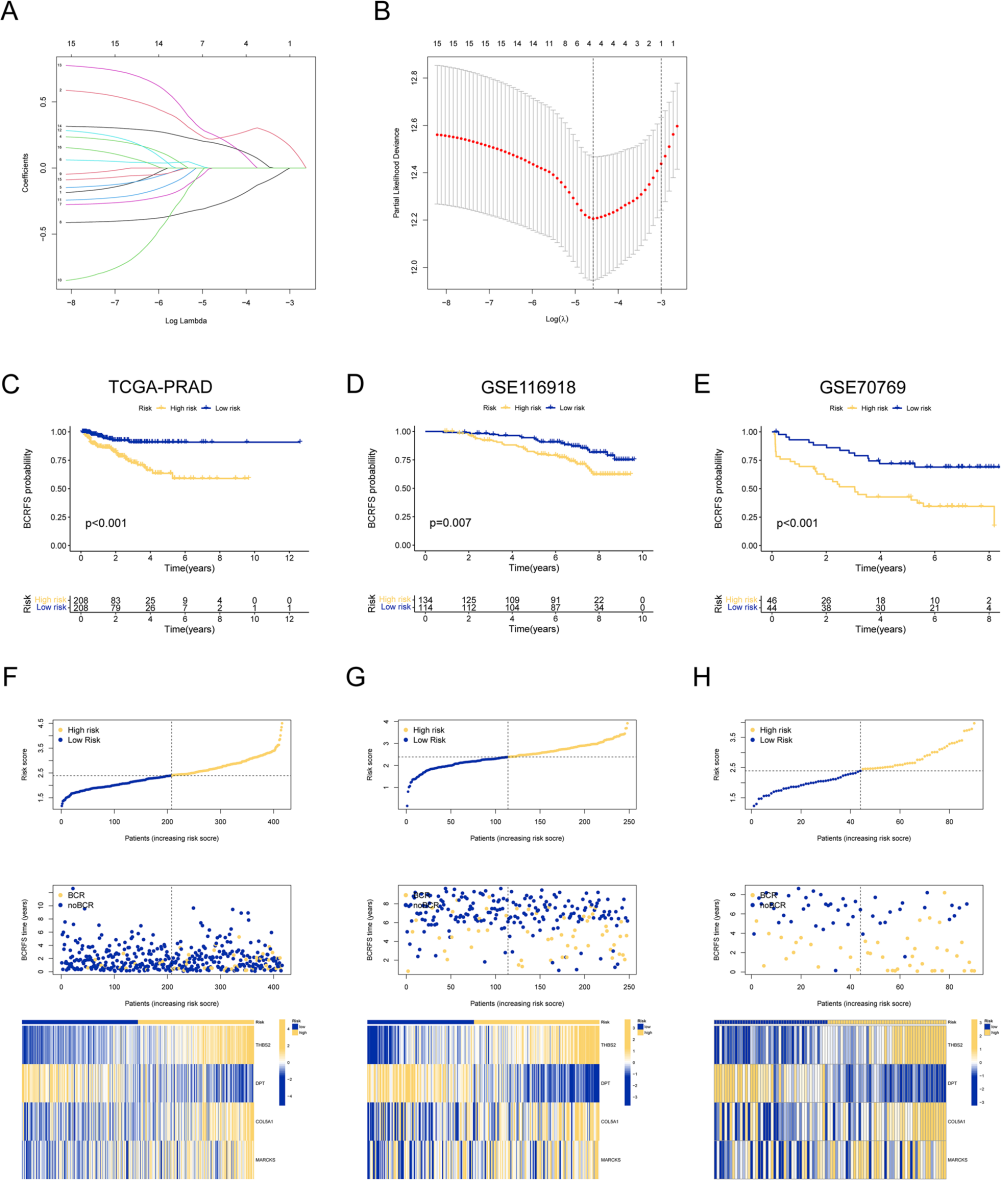
Establishment and validation of a prognostic model based on APPCAF-related genes. **A** The coefficients in Lasso regression analysis. (B) Selection of lambda in the Lasso regression model using tenfold cross-validation. The KM survival curves for the patients from TCGA-PRAD (**C**), GSE116918 (**D**), and GSE70769 (**E**). The risk scores, BCRFS status, and gene expression levels in TCGA-PRAD (F), GSE116918 (**G**), and GSE70769 (**H**)

To establish the APPCAF-related signatures, we initially employed Lasso regression and conducted tenfold cross-validation using the 20 APPCAF-related genes (Fig. 8A, B). Four key genes, THBS2, DPT, COL5A1, and MARCKS, were identified and incorporated into the development of the prognostic model. The TCGA-PRAD cohort served as the training set, whereas the GSE116918 and GSE70769 cohorts served as the validation sets. Using the median signature risk score as a threshold, we classified patients in both the training and validation sets into high-risk and low-risk groups. The KM curve revealed a significant difference in prognosis between the high-risk and low-risk groups, with the former exhibiting worse outcomes (Fig. 8C–E). Furthermore, Fig. 8F–H revealed that higher risk scores were associated with an increased likelihood of BCR occurrence in prostate cancer patients.

### Identification of independent prognostic factors and construction of the nomogram

**Fig. 9:**
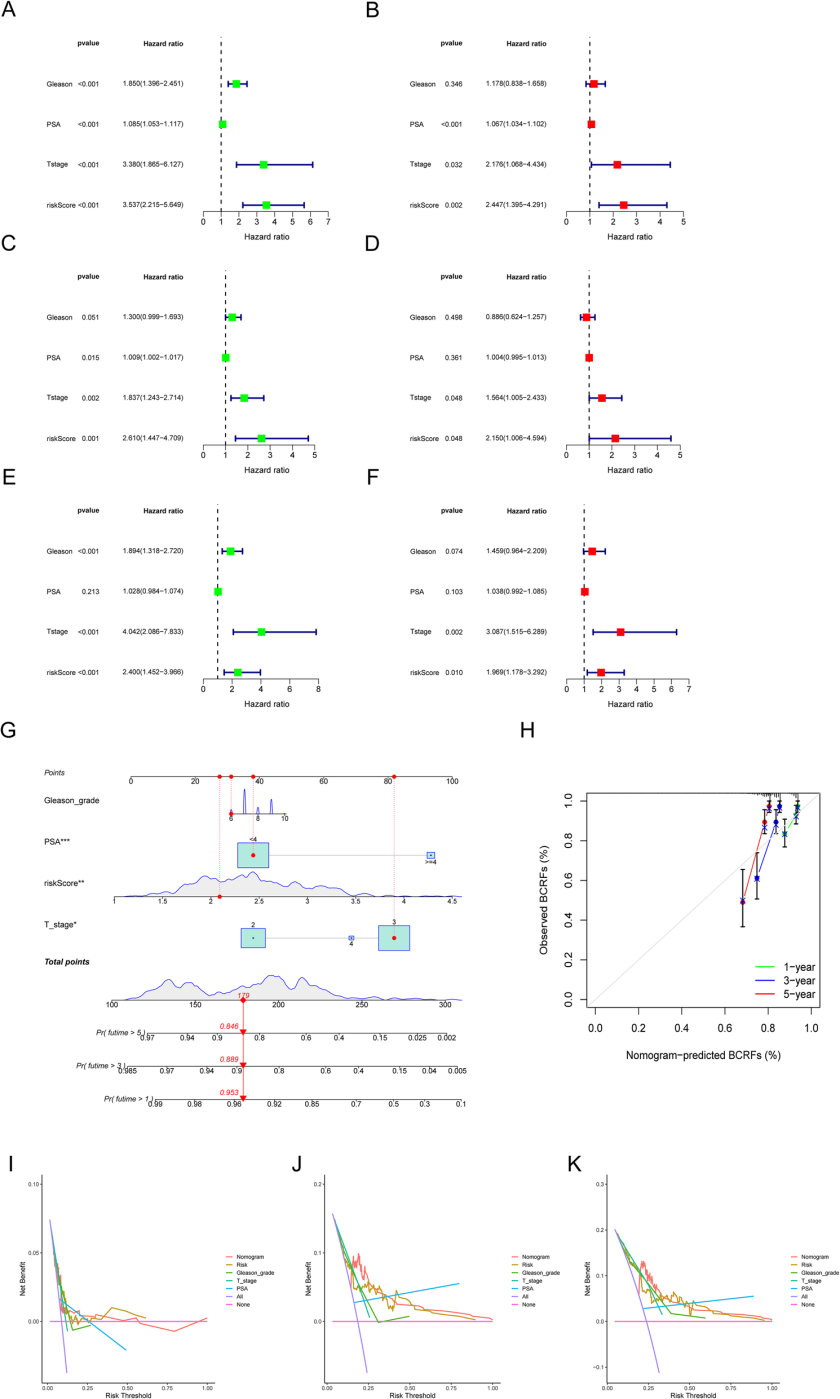
Construction of the nomogram and the prediction accuracy based on independent prognostic factors. Univariate and multivariate regression analyses revealed a significant correlation between BCRFS and risk scores, as well as various clinical parameters in the TCGA-PRAD (**A**, **B**), GSE116918 (**C**, **D**), and GSE70769 (**E**, **F**) cohorts. **G** Nomogram for predicting 1-year, 3-year, or 5-year BCRFS time in PCa patients. **H** 1-, 3-, and 5-year nomogram calibration curves of the TCGA-PRAD cohort Decision curve analysis for 1-year (**I**), 3-year (**J**), and 5-year (**K**) BCRFS

To further assess the predictive performance of our constructed prognostic model for BCR in PCa, we investigated the contribution of various indicators, including Gleason grade, PSA, T stage, and risk score, using univariate and multivariate Cox regression analyses in patients from the TCGA-PRAD, GSE116918, and GSE70769 cohorts. As depicted in Fig. 9A, B, the risk score emerged as an independent prognostic factor for BCR in TCGA-PRAD patients (HR: 2.447, p = 0.002). Similarly, in the GSE116918 cohort, the risk score also demonstrated an independent prognostic value for BCR (HR: 2.150, p = 0.048) (Fig. 9C, D). Moreover, in PCa patients from the GSE70769 cohort, the risk score was identified as an independent prognostic factor for BCR (HR: 1.969, p = 0.010). Subsequently, we developed a nomogram based on Gleason grade, PSA, T stage, and risk score (Fig. 9G). Furthermore, calibration curve analysis indicated a high level of concordance between the predicted BCRFS and the actual BCRFS (Fig. 9H). Decision curve analysis revealed that the nomogram and risk score were more stable and accurate in predicting BCR than Gleason grade, PSA, and T stage (Fig. 9I).

### Correlation between the signatures of APPCAF-related genes and clinical characteristics

**Fig. 10:**
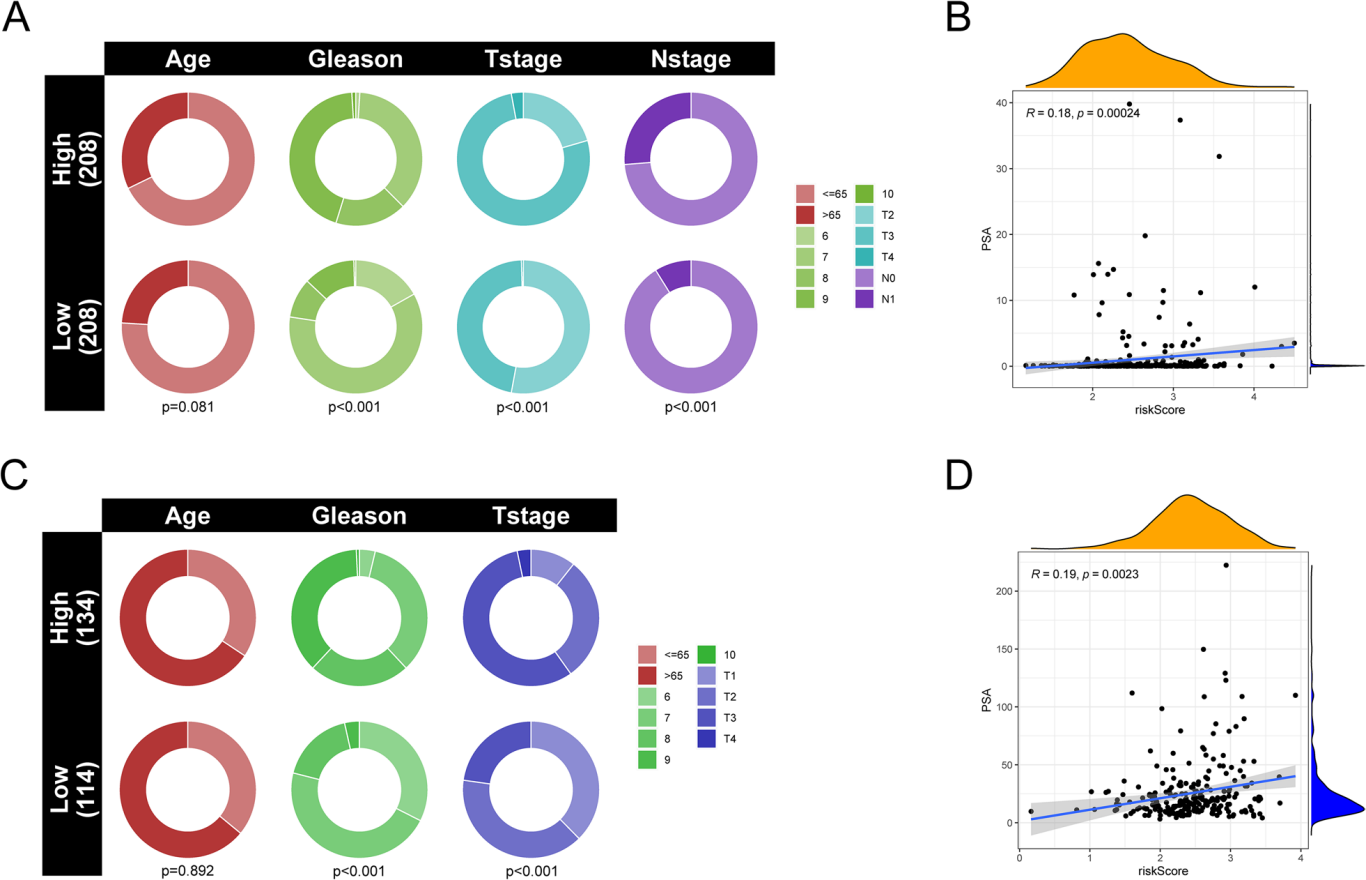
Correlation analysis between risk scores and clinical features. **A** Pie chart depicting the variations in clinical pathological factors between the high-risk and low-risk subtypes in TCGA. **B** Scatter plot illustrating the correlation between risk score and PSA levels in TCGA. **C** Pie chart depicting the variations in clinical pathological factors between the high-risk and low-risk subtypes in GSE116918. **B** Scatter plot illustrating the correlation between risk score and PSA levels in GSE116918

We further evaluated the differences in clinical characteristics between high-risk and low-risk prostate cancer patients. In the TCGA-PRAD cohort, the composition differences in clinical features between the high-risk and low-risk subgroups revealed significant disparities in the Gleason grade, T stage, and N stage (Fig. 10A). Figure 10B illustrates a significant positive correlation between the risk score and PSA (R = 0.18, p = 0.00024). Furthermore, in the GSE116918 cohort, significant differences were observed between the high-risk and low-risk groups in terms of Gleason grade and T stage (Fig. 10C). Additionally, there was a significant positive correlation between the risk score and PSA (R = 0.19, p = 0.0023) (Fig. 10D).

### APPCAFRS-based immune-related discrepancies in PCa

**Fig. 11:**
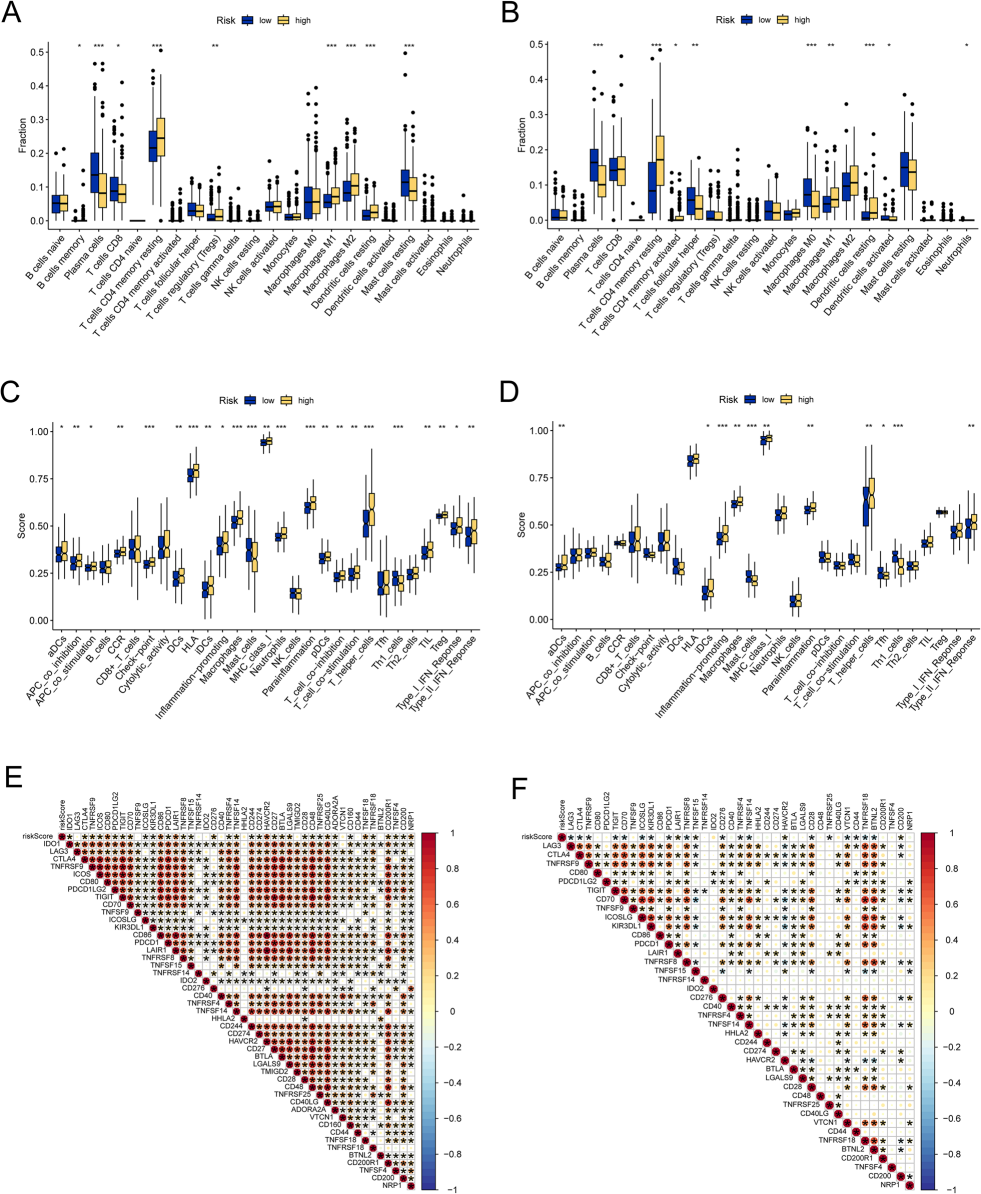
Differential analysis of tumor-infiltrating immune cells between the two risk subgroups. The discrepancy in immune cell infiltration in the two risk subpopulations in TCGA-PRAD (**A**) and GSE116918 (**B**). Comparison of immune function scores between the low and high groups in TCGA-PRAD (**C**) and GSE116918. The correlation between the APPCAFRS-based risk score and immune checkpoint genes in TCGA-PRAD (**E**) and GSE116918 (**F**)

In light of the disparities in the tumor immune microenvironment between the low-risk and high-risk subgroups, we conducted a comprehensive investigation into the immune-related differences in the TCGA-PRAD and GSE116918 cohorts. Initially, a differential analysis of immune cell infiltration between the low-risk and high-risk subgroups in both cohorts revealed an increased abundance of resting memory CD4 + T cells, M1 macrophages, and resting dendritic cells in the high-risk subgroup, while plasma cells exhibited a lower infiltration abundance (Fig. 11A, B). Subsequently, we calculated immune functional scores for patients in both cohorts. As illustrated in Fig. 11C and D, the high-risk group displayed significantly elevated scores for inflammation-promoting, MHC Class I (MHC I), and T helper cells. Additionally, an examination of the correlation between the risk score and immune checkpoint-related genes revealed a negative association between the risk score and multiple immune checkpoint-related genes (Fig. 11E, F). In addition, the analysis of immune therapy sensitivity prediction in low-risk and high-risk patients using the TIDE database revealed that high-risk patients exhibited a diminished response to immune therapy and a greater likelihood of immune evasion (Additional file 4: Figure S1A, B). Furthermore, using the previously established pancancer genomic instability features [37], we analyzed immune subtypes within the low-risk and high-risk subgroups. As depicted in Additional file 4: Figure S1C, high-risk patients demonstrated a higher prevalence of the C1 subtype, which scored higher in tumor mutation burden, noninteger copy number alterations, and homologous recombination deficiency. Finally, in the IMvigor 210 cohort, we stratified patients based on the risk score, and in alignment with our previous findings, patients in the high-risk group exhibited a worse prognosis (Additional file 4: Figure S1D).

### Prediction of chemotherapy sensitivity

**Fig. 12:**
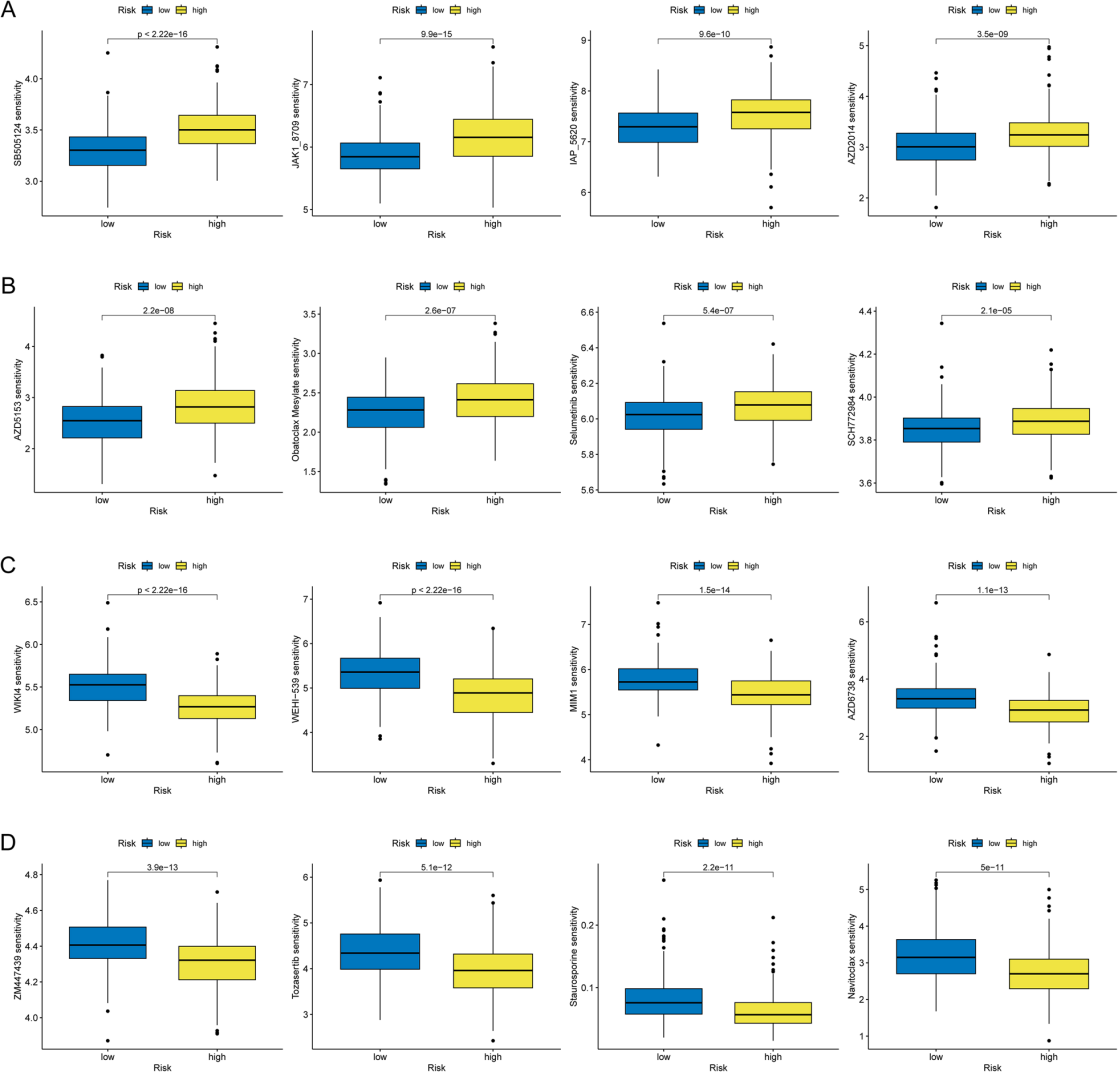
Drug sensitivity prediction in low- and high-risk groups. Box plots depicting more sensitive drugs in the high-risk group (**A**, **B**). Box plot illustrating more sensitive drugs in the low-risk group (**C**, **D**)

To further elucidate the differences in chemotherapy drug response between the low-risk and high-risk subgroups, we evaluated the predictive capacity of the risk model for chemotherapy drug sensitivity using the TCGA-PRAD dataset. Our analysis revealed that the high-risk group exhibited increased sensitivity to eight chemotherapy drugs, including SB505124 and JAK1_8709 (Fig. 12A, B). Conversely, the low-risk group demonstrated higher sensitivity to drugs such as WIKI4 and WEHI-539 (Fig. 12C, D).

### Validation of the feature genes and CD4 + T-cell early activation assay

**Fig. 13:**
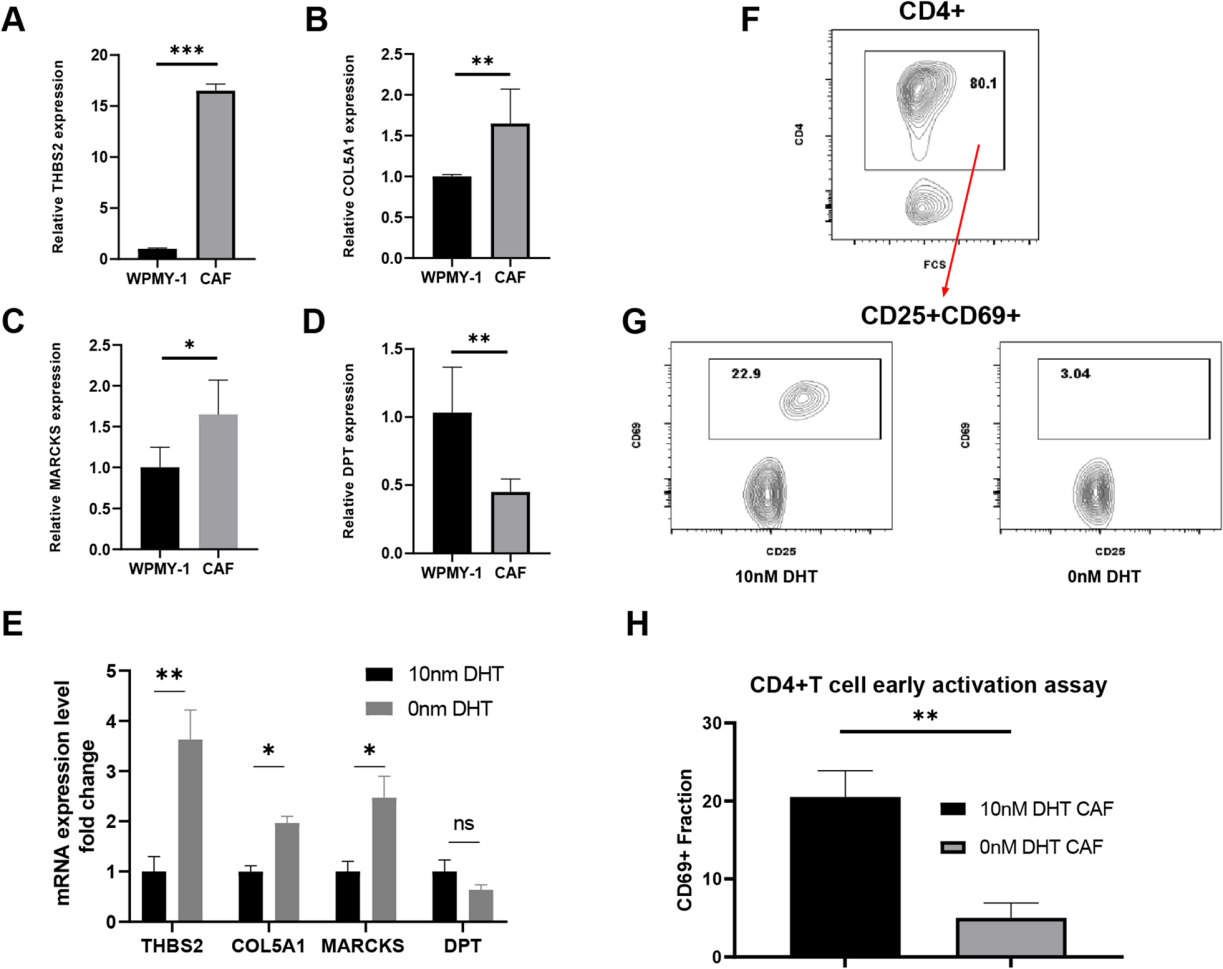
Validation of relative gene expression levels in vitro for genes included in the APPCAFRS. The relative expression levels of THBS2 (**A**), COL5A1 (**B**), MARCKS (**C**), and DPT (**D**) in WPMY-1 cells and CAFs. The relative expression levels of THBS2, COL5A1, MARCKS, and DPT in pre-ADT and post-ADT CAFs (**E**). An example of a T-cell early activation assay. CD4 + T cells that were cocultured with different CAF subtypes. After exclusion by forward- and side-scatter, residual viable cells were stained for CD4, CD25 and CD69. A representative example of CD4 gating is shown in the left Panel (**F**). Each of the other panels shows the CD69 + population upon coculture of T cells with pre-ADT CAFs or post-ADT CAFs following incubation with OVA (G, H)

We conducted qRT‒PCR analysis to investigate the relative differential expression of these signature genes in vitro. The prostate CAFs exhibited a significant upregulation of THBS2, COL5A1, and MARCKS, while DPT showed a significant downregulation when compared to the normal prostate stromal fibroblast line WPMY-1 (Fig. 13A–D). Subsequently, we examined the expression of these signature genes in the pre-ADT and post-ADT groups by subjecting the CAF cell lines to in vitro androgen deprivation therapy. As depicted in Fig. 13E, following ADT treatment, THBS2, COL5A1, and MARCKS were significantly upregulated, whereas DPT showed an insignificant downregulation. Using an early activation assay, compared to pre-ADT CAFs, post-ADT CAFs did not induce measurable OVA-specific T-cell activation in coculture with T cells, as indicated by early activation markers of TCR ligation (CD25 and CD69) (Fig. 13F–H).

## Materials and methods

### Isolation and transcriptome sequencing of primary CAFs

Tumor tissues were collected from patients with prostate cancer who underwent radical prostatectomy at the Department of Urology, Shanghai General Hospital, Shanghai Jiao Tong University School of Medicine. The methodology and procedures for isolating primary CAFs from prostate cancer tissues were previously described [38]. A total of 12 primary CAF samples were obtained from 12 patients. Among them, 6 patients did not receive any treatment within the 3–6 months before radical prostatectomy, while the remaining 6 patients underwent androgen deprivation therapy with abiraterone and leuprorelin before the surgery. Subsequently, RNA sequencing analysis was conducted on 6 pre-ADT and 6 post-ADT primary CAFs using the switching mechanism at the 5' end of RNA template (SMART) technology.

### DEG analysis and enrichment analysis

The DEG analysis was performed on the SMART sequencing results using the "Limma" package [39]. The selection criteria for DEGs were set as |logFC|> 1 and p value < 0.05. Subsequently, we conducted an enrichment analysis on the DEGs by utilizing the GO and KEGG databases.

### Data collection and processing

The scRNA-seq data utilized in this study were obtained from the GSE141445 dataset available in the GEO database [40]. After an initial integration of the samples, we generated gene expression and phenotype matrices consisting of 36,424 scRNA-seq datasets. Additionally, bulk RNA-seq data with clinical characteristics were downloaded from both the TCGA database and the GEO database. The study encompassed three distinct cohorts, namely, TCGA-PRAD, GSE116198 [41], and GSE70769 [38]. To ensure robust data quality, we selected patients with clearly defined biochemical recurrence outcome information, considering a minimum follow-up duration of 30 days. The final analysis included a total of 419 samples from TCGA-PRAD, 248 samples from GSE116918, and 90 samples from GSE70769.

### Visualization of major cell types and subtypes in PCa

Using the "Seurat" package [42], a Seurat object was generated based on scRNA-seq data from the GSE141445 dataset. Initially, cells with gene expression exceeding 4000 or below 200, as well as cells with high levels of mitochondrial gene expression (pctMT > 15%), were excluded. Subsequently, the top 2000 variable genes were selected for data normalization using the FindVariableFeatures function in the Seurat package. We applied the ScaleData and RunPCA functions to the normalized data for principal component analysis (PCA). Dimensionality reduction and visualization of the data were achieved using the t-distributed stochastic neighbor embedding (t-SNE) and uniform manifold approximation and projection (UMAP) methods. Finally, cell annotation and visualization of major cell types or subtypes were conducted based on the expression of specific marker genes for different cell types.

### Nonnegative matrix factorization (NMF) of APPRGs in CAFs

A gene set comprising 431 antigen processing and presentation-related genes (APPRGs) was obtained from the InnateDB database (https://www.innatedb.com) (Table S1). To further investigate the CAFs involved in antigen processing and presentation, we employed the NMF algorithm [43] to perform dimensionality reduction analysis on the 431 APPRGs in CAFs. Different cell types within CAFs were determined based on the scRNA expression matrix.

### Identification of the DEGs and characteristics of APP-related CAF subtypes in PCa

Differential gene analysis was conducted using the FindAllMarkers function, employing filtering criteria of |logFC|> 1 and adjusted p value < 0.05, to identify genes that exhibited significant differences between CAF subtypes, thus warranting further investigation. Subsequently, the CellChat package [44] was utilized to infer and analyze intercellular communication. The netVisual circle function was employed to visualize the strength of cell‒cell communication networks between APP-related CAF subtypes, ranging from the source cell cluster to various other cell clusters. Finally, the AddModuleScore function was applied to calculate feature scores for the APP-related CAF subtypes based on their characteristic genes.

### NMF clustering identification of subtypes of APPCAF-related genes

The correlation between APPCAF-related genes and BCR was evaluated in TCGA-PRAD samples using univariate Cox regression analysis. Subsequently, the mRNA expression matrix of APPCAF-related genes was collected from the TCGA-PRAD dataset. The "NMF" package in R was utilized, and the Brunet method was applied to perform NMF clustering. We determined the optimal value of k, representing the number of clusters, by considering cophenetic correlation coefficients and silhouette scores.

### Analysis of the characteristics of clusters based on APPCAF-related genes

Differential expression analysis was conducted on distinct clusters of APPCAF-related genes in TCGA-PRAD samples using the "limma" package. Subsequently, the ssGSEA function in the “GSVA” package was utilized to evaluate the disparities in immune cell infiltration among the clusters of APPCAF-related genes [45]. Additionally, the GSVA package was employed to perform GO and KEGG enrichment analysis on the DEGs identified between the clusters of APPCAF-related genes.

### Development and validation of a risk signature based on APPCAF-related genes

To mitigate overfitting, we employed the "glmnet" package to perform LASSO regression analysis. Utilizing the APPCAF-related genes associated with prognosis, we constructed an APPCAF-related signature (APPCAFRS). Subsequently, we utilized the "predict" function in R to assign risk scores to each sample in the TCGA-PRAD cohort. Based on the median risk score, the samples were categorized into low-risk and high-risk subgroups. Moreover, employing the median risk score derived from the TCGA-PRAD cohort, we stratified PCa patients from the GSE116918 and GSE70769 cohorts into high-risk and low-risk subgroups. Finally, we conducted the following analyses in the three PCa cohorts: (1) Kaplan‒Meier analysis was performed to assess the survival differences between the high-risk and low-risk subgroups; (2) the "pheatmap" package was utilized to visualize the expression levels of genes in the APPCAF-related signature and the distribution of outcomes in the cohorts; and (3) univariate and multivariate Cox regression analyses were employed to evaluate the independence of the risk score and clinical features as prognostic factors.

### Establishment of a nomogram and comparison of clinical features between low- and high-risk patients

To predict the 1-year, 3-year, and 5-year biochemical recurrence-free survival (BCRFS) in PCa patients, a nomogram was developed. Subsequently, to assess the performance of the nomogram, a calibration curve was generated by comparing the predicted probabilities with the observed outcomes at 1 year, 3 years, and 5 years, thereby gauging its accuracy. Additionally, a chi-square test was conducted to examine the associations between the APPCAF-related signature (APPCAFRS) and clinical features such as T stage, Gleason grade, and PSA level.

### Investigation of immune-related differences based on the APPCAF-related signature

To explore the immune-related differences associated with the APPCAFRS, we examined the variations in the tumor immune microenvironment between low-risk and high-risk subgroups. Initially, the CIBERSORT algorithm was applied to calculate the infiltration composition of 22 immune cell types in each PCa sample, allowing for an analysis of the changes in immune cell infiltration between the low-risk and high-risk subgroups [46]. Subsequently, the activity differences of immune-related pathways between the high-risk and low-risk subgroups were assessed using the ssGSEA function in the "GSVA" package, with the Wilcoxon rank-sum test employed for analysis.

### Exploring immunotherapeutic responsiveness and potential drug treatments

To pinpoint patients who could potentially benefit more from immune checkpoint inhibitor (ICI) therapy, we conducted a correlation analysis between the risk score and immune checkpoint-related genes. Subsequently, the Tumor Immune Dysfunction and Exclusion (TIDE) tool was applied to assess the immune treatment response in prostate cancer patients, evaluating the responsiveness to immune therapy between the high-risk and low-risk subgroups using a chi-square test. Moreover, we utilized the "oncoPredict" package to predict drug sensitivity between the high-risk and low-risk subgroups.

### Cell culture

The human benign prostate stromal cell line (WPMY-1) was obtained from the Cell Bank of the Shanghai Institutes for Biological Sciences, Chinese Academy of Sciences. WPMY-1 cells were cultured in DMEM containing 5% fetal bovine serum and 1% penicillin/streptomycin. All cells were cultured at 37 °C in a 5% CO2 environment.

### Validation of feature gene expression by in vitro qRT‒PCR

To further validate the APPCAFRS, we conducted qRT‒PCR analysis to assess the expression of characteristic genes in normal prostate stromal fibroblasts (WPMY-1), prostate cancer-associated fibroblasts (hTERT PF179T CAF), and pre-ADT and post-ADT CAFs. The ADT of CAFs was performed following a previously described method [47]. RNA was extracted from the cells using TRIzol reagent (Takara, Japan), and cDNA was synthesized using a reverse transcription kit (Vazyme, China). Real-time PCR was carried out using the SyberGreen method to quantify the expression of target genes. The primer sequences utilized in this study can be found in Table S2.

### CD4 + T-cell early activation assay

After primary CAFs were sorted, 10 nM DHT and 10 nM ETOH were applied to simulate androgen intervention and cultured in vitro. A total of 1250–2500 sorted CAFs were cultured with 25 μg/ml OVA peptide 323–339 or without a peptide in U-bottom 96-well plates and incubated at 37 °C and 5% CO2. PBMCs corresponding to primary CAFs were used to isolate and enrich CD4 + naïve T cells using the MojoSort Human CD4 Naïve T-Cell Isolation Kit (Biolegend #480,041). The CAF plates were washed twice, and 2500 CD4 + T cells were cocultured with 10% FBS/DMEM per week for 17 h. Cells were then washed and blocked and stained with the following antibodies (all from Biolegend at 1:200): CD4 (Clone RPA-T4), CD25 (Clone PC61.5), and CD69 (Clone FN50) for 30 min at 4 °C.

## Discussion

Growing evidence suggests that the malignant biological behaviors of tumor cells are dependent on the intercellular communication between tumor and stromal cells in a complex microenvironment [16–18, 48]. As essential components of the TME, CAFs regulate tumor proliferation, angiogenesis, invasion, metastasis, and treatment resistance in numerous malignancies [49, 50]. With recent advancements in cancer research, there are numerous approaches to overcome drug resistance, among which immunotherapy has revolutionized cancer treatment [51, 52]. Evidence suggests that antigen-presenting cells are essential for T-cell activation and tumor immunity and that cancers can circumvent this immunity through means of immune editing, such as immune dominance, the absence of immune checkpoints, or downregulating antigen-presenting cells [53, 54]. Antigen-presenting cells are crucial for launching, programming, and regulating tumor-specific immune responses [55, 56]. ApCAFs, a novel type of cancer-associated fibroblast capable of presenting MHC II-mediated antigens within the TME, were recently identified in pancreatic ductal adenocarcinoma and breast cancer [26, 57].

In this study, we sorted CAFs from radical prostatectomy samples of patients who received neoadjuvant therapy and patients who did not receive neoadjuvant therapy within 3–6 months. We discovered that the expression level of MHC-II-related molecules (HLA-DQA1, HLA-DRB1, and HLA-DRA) was significantly reduced in CAFs after neoadjuvant treatment, as were the corresponding functional pathways (MHC class II protein complex assembly, antigen processing, and presentation of peptide antigen via MHC class II). We focused on specific CAFs associated with antigen presentation and systematically characterized and classified CAFs in PRAD using scRNA-seq data. Ultimately, we identified the CTSK + MRC2 + CAF cluster as an APPCAF cluster that interacts strongly with T cells, which may help regulate different aspects of tumor immune microenvironment (TIME) biology. Capitalizing on the unique attributes of these CAF cells, precise modulation of the tumor microenvironment is feasible, thus enhancing the post-ADT antitumor immune profile in PCa patients. Furthermore, we devised a predictive model for biochemical recurrence in PCa patients. This model not only holds substantial promise for biomedical applications but also facilitates accurate stratification of PCa patients at an early stage, consequently elevating long-term prognostic outcomes.

Expanding upon the groundwork of our preliminary research, it is evident that the antigen presentation and processing functions of CAFs within the prostate cancer tissue microenvironment tend to wane following ADT treatment, potentially facilitating immune evasion. Consequently, directing interventions toward CAFs equipped with antigen presentation capabilities could hold significant promise in benefitting patients with prostate cancer. Against the backdrop of flourishing advancements in novel biomaterials and nanotechnology, more refined targeted nanoparticle systems have been meticulously devised for efficient drug delivery. Conventional inorganic nanomaterials, including metal nanoparticles and carbon-based counterparts, have been associated with inherent neurotoxicity [58]. Conversely, green nanomaterials are surfacing as a groundbreaking avenue, boasting reduced toxicity [59]. The research conducted by Mousavi et al. substantiates that environmentally friendly synthesized silver nanoparticles induce apoptosis and unveil dose- and time-responsive cytotoxic as well as anticancer effects on gastric cancer cells [60]. Moreover, Patrascu et al. elucidated the efficacy of a hybrid nanosystem comprising biopolymeric membranes and silver nanoparticles, manifesting pronounced cytotoxicity against murine fibroblast L929 cells [61]. Our enthusiastic outlook is grounded in the convergence of biomaterials and biomedicine directed at CAFs and strategic intervention in the intricate tumor microenvironment—a burgeoning domain of research.

Increasing evidence has confirmed the prognostic value or therapeutic prediction of CAF-related gene markers in PRAD [62]. Based on the prognostic value of the CTSK + MRC2 + CAF cluster, we identified the differentially expressed genes between the CTSK + MRC2 + CAF cluster and other CAF clusters and further developed an APPCAF-based risk signature with 4 genes; it was composed of one protective gene (DPT) and three risk genes (THBS2 COL5A1 MARCKS). Among these four genes, MARCKS had the highest CNV loss frequency. CNV mutation burden affects gene expression level or activity, thereby influencing genetic modulation and causing PRAD progression [63, 64]. We further clarified the differentiation ability of APPCAF signature genes for prostate cancer via NMF clustering, which suggests that patients in APPRG Cluster C2 are associated with poorer clinical outcomes, and we discovered that prostate cancer samples with varying APPRG expression levels were significantly correlated with various pathways. APPRG Cluster C2 samples were significantly associated with mismatch repair, DNA replication, and somatic diversification of immunoglobulins, while APPRG Cluster C1 samples were significantly associated with fatty acid metabolism, glutathione metabolism, and linoleic acid metabolism. Previous studies demonstrated a significant correlation between microsatellite instability or mismatch repair status and the efficacy of immune checkpoint inhibitors in prostate cancer [65–67]. Despite being uncommon, immune checkpoint blockades are effective for those with advanced prostate cancer and mismatch repair gene mutations [68].

CAFs interact intimately with immune T cells in the TME, thereby promoting the progression of the tumor [69, 70]. Sample risk exhibited a significant positive correlation with CD4 + T cells and a significant negative correlation with CD8 + T cells in our signature. In addition, a negative association was found between a high-risk score and the expression level of immune checkpoint inhibitor target genes. The TIME is composed of numerous and diverse immune cells in tumor tissues and significantly influences the immune status of the TME, thereby influencing the immunotherapy efficacy of patients [71–73]. As essential components of the TME, CAFs can interact directly with immune infiltration and remodel the immunosuppressive TME, allowing tumor cells to evade immune surveillance [74–76]. Despite the paucity of research on CAFs, Elyada and Friedman et al. demonstrated the effect of CAFs on the TIME via the antigen presentation method [26, 57]. In addition, apCAFs can trigger the local activation of CD4 + T cells and induce memory. Kerdidani et al. demonstrated that apCAFs were also capable of activating tumor-specific CD4 T cells and recruiting near CD4 T cells both in vivo and in vitro.

Concurrently, in previous studies, CAFs were preferable for activating tumor cells and delivering microRNAs or other substances to tumor cells after androgen deprivation [26, 47, 77]. Few studies considered the changes in the immune characteristics of CAFs before and after castration to study their effect on tumor cells. In regards to novelty, we demonstrated for the first time that androgen withdrawal treatment leads to a decline in antigen presentation and a process-related phenotype in prostate CAFs. This observation is pivotal, as it holds the potential to amplify the effectiveness of ADT in patients with prostate cancer. Based on the above clues, we further identified a potential CAF subtype in the prostate associated with antigen presentation and processing at the single-cell level. Similar phenotypic features of CAFs have been previously reported in pancreatic cancer [35] but not in prostate cancer. Notably, no antecedent research within the domain of the prostate cancer immune microenvironment has hitherto singled out antigen presentation and processing-associated CAFs. Furthermore, a novel signature related to APPCAF in prostate cancer was established. Compared with previously published CAF-associated prostate cancer signatures [61, 78], our signature is more reliable and clinically instructive, based on clinical specimens and derived from a group of potential CAF subtypes. Meanwhile, our findings revealed that the APPCAF-based signature had predictive potential for both prognosis and treatment response. These findings shed new light on the role of APPCAFs in the remodeling of tumor niches and the immune status of the TME.

However, our study also has several limitations. The generation of APPCAF clusters and APPCAF-based risk signatures was accomplished using retrospective data obtained from a public database. To avoid selection bias and enhance the accuracy of the analysis, future validation of this signature will require more prospective and multicenter PRAD cohorts. In addition, we only evaluated the APPCAF-based risk signature in predicting prognosis. Therefore, our next objective is to conduct a comprehensive study aimed at elucidating the potential mechanisms underlying this signature, with the ultimate goal of its clinical administration.

## Conclusions

We used a comprehensive bioinformatics analysis to identify DEGs between patients who received or did not receive ADT and discovered that CAFs downregulate the activity of antigen presentation and process-related pathways after castration. CTSK + MRC2 + CAF-C1 was identified as a CAF subtype associated with potential antigen presentation and processing. A signature based on four APPCAFRGs (THBS2, DPT, COL5A1, and MARCKS) was developed and validated, and the risk score derived from the signature demonstrated an inverse correlation with the infiltration of various immune cells, indicating that high risk was significantly correlated with poorer prognosis and clinical outcomes in PRAD. In vitro experiments were conducted to confirm the expression levels of four APPCAFRGs. These findings contribute to a better comprehension of the causes of immunotherapy's poor efficacy in prostate cancer. Meanwhile, the study and investigation of the CAF subgroup of prostate cancer are aimed at exploring the relevant characteristics of antigen presentation and the process of CAFs in PRAD and offer new avenues for exploring potential combination treatment strategies.

### Supplementary Information

**Additional file 1: Table S1.** Gene list of  antigen processing and presentation-related genes (APPRGs).

**Additional file 2: Table S2.** Primer information of qPCR.

**Additional file 3: Table S3.** 55 DRGs of the CTSK + MRC2 + CAF-C1 and NoneAPP-CAF-C2 subgroups.

**Additional file 4: Figure S1.** Comparison of immunotherapy and immune subtypes in the high-risk and low-risk groups. Immune treatment responsiveness and TIDE scores analyzed between high-risk and low-risk groups in TCGA-PRAD (A) and GSE116918 (B). (C) Immunosubtype analysis based on risk scores conducted in the TCGA samples. (D) Based on APPCAFRS, overall survival was analyzed in the IMvigor210 cohort.

## Data Availability

The data used to support the findings of this study are available from the corresponding author upon request.
